# Optimized design of SynRM drive systems for high-efficiency solar water pumps

**DOI:** 10.1016/j.heliyon.2024.e39478

**Published:** 2024-10-22

**Authors:** Gullu Boztas, Omur Aydogmus, Musa Yilmaz

**Affiliations:** a Department of Electrical and Electronics Engineering, Faculty of Technology, Firat University, Elazig 23200, Turkey; b Department of Mechatronics Engineering, Faculty of Technology, Firat University, Elazig 23200, Turkey; c Bourns College of Engineering, Center for Environmental Research and Technology, University of California at Riverside, Riverside, CA 92521, USA; d Department of Electrical and Electronics Engineering, Batman University, Batman, 72100, Turkey

**Keywords:** High efficiency, Irrigation, Solar water pump, Solar energy, Synchronous reluctance motor

## Abstract

This study presents the design and implementation of a Synchronous Reluctance Motor (SynRM) with an integrated drive circuit for a 4-inch submersible pump motor, tailored for small-scale solar photovoltaic water pumping systems. The SynRM operates efficiently at low voltage levels, eliminating the need for a boost converter and allowing direct connection to low-voltage power sources. With a power output of 0.55 kW and an ultra-premium efficiency rating of 86.5%, the motor integrates the drive circuit within its casing for a compact design. It operates across a voltage range of 15 V to 95 V, using efficient buck converters for required output voltages. This paper discusses the design, optimization, and prototyping of the motor and drive circuit, highlighting innovative solutions for advancing small-scale solar-powered water pumping systems. Electric motors, especially pump motors, are major consumers of electrical energy. Enhancing their efficiency can significantly impact total energy consumption. This study's SynRM and integrated drive system, optimized for high efficiency, durability, and cost-effectiveness, can be directly powered by PV panels, offering a compact and efficient alternative to traditional systems.

## Graphical abstract

**Figure:**
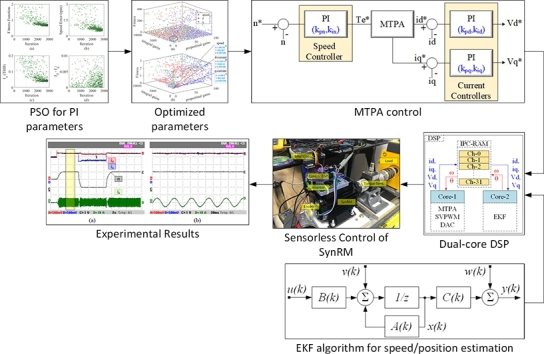

## 1 Introduction

Electric motors account for nearly half of the world's total electricity consumption [1]. Most of these motors are used for pump and fan applications [2], with the majority of pump applications being for irrigation purposes [3], [4], [5]. Irrigation, essential for agriculture, is increasingly relying on solar water pumping units as a sustainable alternative to diesel-based systems, addressing high fuel costs and strict emission laws, with detailed procedures for sizing and economic assessment provided for their effective implementation [6], [7]. Water pumping systems are crucial for irrigation [8], and using solar energy for this purpose offers significant advantages, particularly in areas without electricity, where electricity costs are high, or in remote locations [9]. Many regions around the world have both solar irradiation and groundwater available, making solar energy increasingly popular for small-scale irrigation systems [10]. Submersible pump motors are commonly used in solar photovoltaic (PV) water pumping systems [11], [12], [13]. These low-power motors can be connected directly to PV panels without the need for batteries or boost converters, resulting in a more compact design [14]. Some manufacturers offer PV-connected submersible pump systems that include a low voltage motor, a pump, and a voltage source inverter, which can be connected directly to PV panels. To maximize power extraction from solar PV panels, maximum power point tracking (MPPT) algorithms are essential. Various MPPT algorithms are discussed in the literature, with researchers comparing their effectiveness [15]. These algorithms are classified into two main categories: conventional and intelligent. Conventional algorithms, such as Perturb and Observe (P&O), Incremental Conductance (InC), and Constant Voltage Controller (CVC), often perform poorly under rapidly changing environmental conditions. In contrast, intelligent algorithms, including those based on artificial intelligence (AI), fuzzy logic (FL), and ANFIS, demonstrate superior performance in such conditions [16], [17].

Induction motors (IM), Brushless Direct Current (BLDC) motors and Permanent Magenet Syncronous Motor (PMSM) are commonly used in solar-powered systems [18], [19], [20]. However, induction motors often struggle to perform well at low voltage levels and typically require a boost converter between the motor terminals and the PV panels to achieve the necessary voltage [21]. Despite this, induction motors generally lack efficiency for low-power applications, usually around a few kilowatts. In contrast, BLDC motors perform well in solar-powered systems when directly connected to PV panels for low voltage applications. To achieve high efficiency, performance, power density, and compactness, BLDC motors use rare-earth magnets. However, the supply of these rare-earth magnets is limited and potentially at risk of depletion [22], [23]. Additionally, the market prices for rare-earth materials such as neodymium and dysprosium have surged dramatically and continue to rise rapidly [24]. The cost of these magnets can represent nearly half of the total motor cost [25], [26]. Consequently, designing affordable motors has become increasingly important for low-power solar water pump systems [27].

AC motors share a common stator structure with three-phase windings, making the rotor structure a crucial factor influencing both performance and cost. The rotor of an electric motor can be designed with either windings or permanent magnets. Alternatively, a motor can be designed using the reluctance torque concept, which eliminates the need for rotor windings or magnets. One such motor is the SynRM, which is based on reluctance torque. Although theoretical studies of SynRM date back to 1923 [40], the motor did not gain popularity initially due to the complexity of its control algorithms [41], [42], [43]. In 1992, Vagati and Fratta introduced a vector control technique for reluctance motors, significantly improving their dynamic performance [44]. With advances in motor control technology, interest in SynRMs has grown. The Field Oriented Control (FOC) technique, proposed by F. Blaschke in the early 1970s for controlling induction motors (IM), has been widely adopted in high-performance AC drives due to progress in power electronics, computers, and microelectronics [45], [46], [47]. Various control techniques have been proposed for SynRM [48], [49], [50], [51], [52], [53]. The rotor of a SynRM can be manufactured using a standard lamination press machine, which simplifies the production process and reduces costs, as it does not require additional materials or complex processing compared to other AC motors. Despite these advantages, commercial SynRMs suitable for solar systems are not yet available on the market. A summary of solar water pumping systems with a focus on electric motors is provided in Table 1.

**Table 1:** Examples in the literature of solar water pumping systems focusing on the electric motor.

Study	Motor Type	Motor Power	Motor Drive Type	Implementation Platform	Focus of study
[27]	SynRM	630 W	Inverter	TMS320F28335	Low voltage level SynRM was optimized and implemented for direct connected solar water pumping system.
[28]	SynRM	630 W	Inverter	TMS320F28335	A SynRM was designed as IE4 Super Premium Efficiency class for low voltage level applications.
[29]	SynRM	1.5 kW	Boost + Inverter	dSPACE-1202	The study focuses on a grid-interfaced solar PV system for water pumping and domestic loads, using a SyRM to ensure efficiency and reliability despite the intermittent nature of solar power.
[30]	SynRM	2.2 kW	Boost + Inverter	dSPACE-1103	An improved control for SyRM-based PV water pumping system was studied for eliminating the speed controller of position sensorless FOC of SyRM.
[14]	PMSM	565 W	Inverter	MATLAB	Direct connected PV solar system with low voltage PMSM was proposed for solar water pumping systems.
[31]	PMSM	2.8 kW	Inverter	dSPACE-1202	A novel single sensor-based MPPT technique in order to decrease the cost and improves the reliability of the solar water pumping system.
[32]	PMSM	2.4 kW	Boost + Inverter	dSPACE-1104	A fuzzy precompensated hybrid PI controller for a PMSM-driven standalone solar water pumping system.
[33]	BLDC	1.3 kW	Inverter	dSPACE-1104	Bidirectional power flow control of a grid interactive solar PV-fed water pumping system with a BLDC motor drive was used to run a water pump.
[34]	BLDC	1 kW	SEPIC + Inverter	MATLAB	A neural network based MPPT for photovoltaic BLDC motor connected water pumping system was proposed using SEPIC and inverter structure together.
[35]	BLDC	1.5 kW	Inverter	TMS320F28069	An sensorless integrated motor drive circuit was proposed for a bore-well submersible pump motor.
[36]	BLDC	1.3 kW	Inverter	dSPACE-1104	A position sensorless BLDC motor-driven solar photovoltaic fed water pump was demonstrated through the hardware implementation.
[37]	IM	430 W	Boost + Inverter	TMS320F28379D	A quadratic V/f control method was introduced to drive an induction motor powered directly from a solar PV source using a two-stage power converter without storage batteries.
[38]	IM	2.2 kW	Inverter	dSPACE-1202	An efficient method for control of a solar water pumping system consisting of an induction motor drive was proposed with a model reference adaptive system based adaptive mechanism of speed estimation.
[39]	BLDC	850 W	Boost + Inverter	N/A	The aim of the study is to evaluate a motor drive system powered by a solar PV array and grid, using a boost DC–DC converter for MPPT and a bridgeless converter to enhance power quality, with performance assessed under various energy sources.

In this study, a SynRM directly connected to photovoltaic panels was designed, optimized, and prototyped for use in a solar photovoltaic water pumping system. Sections 2 through 4 cover the mathematical modeling, control, design, and optimization of the SynRM. Section 5 presents an analysis of the system with solar PV panels. The motor prototyping, which includes an integrated motor drive, and the experimental results are detailed in Sections 6 and 7, respectively.

## 2 Motivation and contributions

In solar-powered PV irrigation systems, the motor types commonly used with low power and directly with solar PV panels are DC motors, IM, BLDC, and PMSM. However, these motors incur additional costs due to the extra materials and processes required for rotor production. Additionally, the complexity of the rotors in these motors increases the risk of failure. Therefore, one of the primary goals of this study is to develop a low-cost, highly reliable rotor design for a SynRM. Since SynRMs do not have magnets or windings in the rotor, their production costs and durability are superior compared to other motor types. Moreover, these motors offer nearly constant efficiency and torque regardless of speed.

Another objective is to develop a low-voltage motor that can operate directly with the PV panel voltage level without requiring an additional voltage boosting process. In current systems, the motor voltage is typically at a standard level, with the PV panel voltage being boosted using a Boost Converter. However, in the proposed system, the motor is designed to match the PV voltage, eliminating the need for such conversion. Designing a motor with low voltage presents a significant challenge, especially since this motor does not create additional magnetic fields compared to other motor types. The reluctance torque effect in the rotor is the only source of torque. Additionally, the risk of core saturation due to high currents in the stator windings from low voltage makes the design more complex. Another goal of this study is to design a motor with ultra-premium efficiency. Achieving high efficiency in small-scale motors is a significant challenge. In this study, the desired motor has been successfully designed and optimized by overcoming all these challenges.

The third goal of this study is to integrate the motor driver into the motor case as an alternative to existing systems. By housing the driver within the motor case, the DC-link voltage produced by the PV panels can be directly used as a +/- supply voltage, which is applied to the motor through a 2-wire conductor structure. This reduces the number of cables from three to two and provides a more advantageous system due to better current-carrying capacity with DC in a single conductor. Additionally, since the submersible pump motor operates in water, it can naturally achieve the desired heat transfer, ensuring better cooling for both the motor and the driver. In summary, this study aims to: achieve a more efficient system, reduce production costs, create a more durable structure, and decrease the number of components.

## 3 Control of synchronous reluctance motor

**Figure 1:**
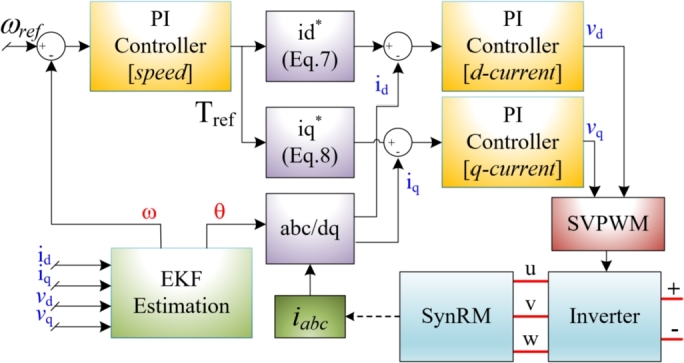
Block diagram of vector control for SynRM.

The vector control approach was used to control the SynRM. The control algorithm derives the *dq*-axes currents $i_{d_{ref}}$ and $i_{q_{ref}}$, which are controlled using a PI controller. The amplitude of the flux linkage can be expressed as given in Eq. (1). These current references are derived from the torque reference, which is obtained from the speed controller, as shown in Fig. 1. The torque expression is provided in Eq. (2).

(1) $$\psi_{s}=\sqrt{{\left(L_{d}i_{d}\right)}^{2}+{\left(L_{q}i_{q}\right)}^{2}}$$

(2) $$T=\frac{3}{2}p\left(\frac{L_{d}}{L_{q}}-1\right)i_{d}\sqrt{\psi_{s}^{2}-{\left(L_{d}i_{d}\right)}^{2}}$$

The constant value of $i_{d}$ can be derived when Eq. (2) is solved using $\partial T/\partial i_{d}=0$ and $\partial T/\partial i_{q}=0$. The equation of $i_{d}$ and $i_{q}$ are given in Eq. (3).

(3) $$i_{d}=\frac{\psi_{s}}{\sqrt{2}L_{d}}\;,\;i_{q}=\sqrt{\frac{\psi_{s}^{2}-{\left(L_{q}i_{q}\right)}^{2}}{L_{d}^{2}}}$$

The $i_{d_{ref}}$ is expressed as given in Eq. (4) when considering the maximum flux. In addition, the ratio of synchronous and rotor speed can be used in the equation for field weakening region.

(4) $$i_{d_{ref}}=\frac{\psi_{maxref}}{\sqrt{2}L_{d}}\frac{\omega_{r}}{\omega_{s}}$$

where $\omega_{r}$ is the rotor angular speed and $\omega_{s}$ is the synchronous angular speed of the motor. The $i_{q_{ref}}$ can be obtained by using Eq. (5).

(5) $$i_{q_{ref}}=\frac{2T_{ref}}{3p\left(L_{d}-L_{q}\right)i_{d_{ref}}}$$

The control strategy depends on the angle *φ* which can be obtained by dividing both currents given in Eq. (3). The angle *φ* is given in Eq. (6).

(6) $$\frac{i_{q}}{i_{d}}=\frac{\psi_{s}/\sqrt{2}L_{d}}{\psi_{s}/\sqrt{2}L_{q}}=\frac{L_{d}}{L_{q}}=tan(\varphi )$$

The final equations can be modified as given in Eq. (7) and Eq. (8) which are used in the control strategy [54].

(7) $$i_{d}^{⁎}=\sqrt{\frac{2|T_{ref}}{3p\left(L_{d}-L_{q}\right)tan(\varphi )}}$$

(8) $$i_{q}^{⁎}=\frac{i_{d}^{⁎}sgn\left(T_{ref}\right)}{tan(\varphi )}$$

**Figure 2:**
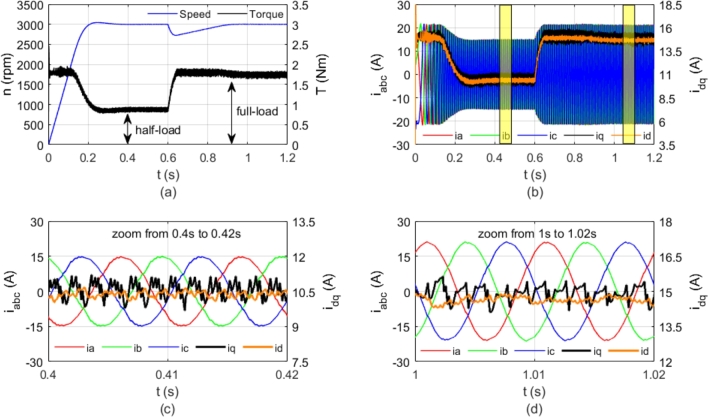
Simulation results; a) Speed and torque, b) Stator currents and dq-axes currents, c) Detail view of figure-b from 0.4 s to 0.42 s, d) Detail view of figure-b from 1 s to 1.02 s.

The use of the equations in the control algorithm is depicted in Fig. 1. In this study, the angle was set to π/4. The control strategy was analyzed using MATLAB/SimScape, with the results shown in Fig. 2. The motor was initially loaded to half-load and subsequently to full-load, operating at 3000 rpm for 1.2 seconds. The speed and torque curves for these conditions are presented in Fig. 2(a). The motor's dynamic response is slow due to the current limitations of the controller. If the angle value were reduced below π/4, the motor current would increase, resulting in a higher current drawn from the PV panels and an instantaneous decrease in the PV panel voltage. Consequently, a low dynamic response with low current draw was preferred over a fast dynamic response due to the pump load. The motor current was limited to 15 A rms, as shown in Fig. 2(b). The stator currents and *dq*-axis currents are also shown in Fig. 2(b). The regions highlighted in yellow in Fig. 2(b) are detailed in Fig. 2(c) and Fig. 2(d). In all cases, the *d*-axis and *q*-axis currents are maintained at equal levels. This control approach is referred to as maximum torque per ampere (MTPA). The sensorless algorithm related to the designed motor is presented in [55]. For comprehensive details on the SynRM's sensorless control, refer to the previous study by the authors of this paper.

## 4 Design and optimization of synchronous reluctance motor

The first step in designing an electric motor is to select the motor type and frame size according to standards set by the National Electrical Manufacturers Association (NEMA) and the International Electrotechnical Commission (IEC). This allows for the outer diameter of the stator to be fixed during the optimization process. In this study, a 4-inch submersible pump motor with a stator outer diameter of 90 mm was used. The number of pole pairs and stator slots were chosen as 2 and 24, respectively. The stator's geometry and the rotor's inner and outer diameters were fixed during optimization. The rotor structure was optimized to achieve the highest possible torque and the lowest torque ripple using a genetic algorithm. The MAGNET and MATLAB programs were used together for the optimization process. A finite element analysis (FEA) with the Newton-Raphson iteration method was employed for motor analysis within the optimization loop. In the design process, the optimization parameters utilized are as follows: The number of design variables is set to 8, with a population size of 90 individuals. Each chromosome is sized to 8 genes, and the algorithm is run for 20 generations. The termination criterion for the generations is set to 100. The crossover fraction is specified as 0.75, and the Pareto fraction is 0.7. The migration range is established at 10, and the mutation function used is “@Adaptation feasible.” These parameters are carefully chosen to optimize the design efficiently.

**Figure 3:**
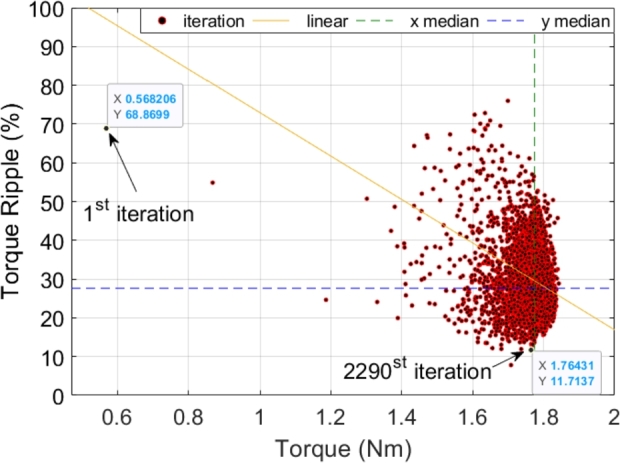
Optimization progress for torque and torque ripple.

The graph in Fig. 3 depicts the optimization progress for torque and torque ripple over numerous iterations. Initially, at the first iteration, the torque is approximately 0.57 Nm with a torque ripple of about 69%. As the optimization progresses through 2290 iterations, the system demonstrates significant improvements, culminating in a torque of around 1.76 Nm and a much-reduced torque ripple of approximately 12%. The scattered red dots represent the iterative data points, showing the gradual convergence towards optimal values. The linear trend line suggests a general decrease in torque ripple as torque increases. Additionally, the green dashed line indicates the median value for torque, and the blue dashed line represents the median for torque ripple. This visualization effectively illustrates the optimization's efficiency in enhancing motor performance by increasing torque while minimizing torque ripple.

**Figure 4:**
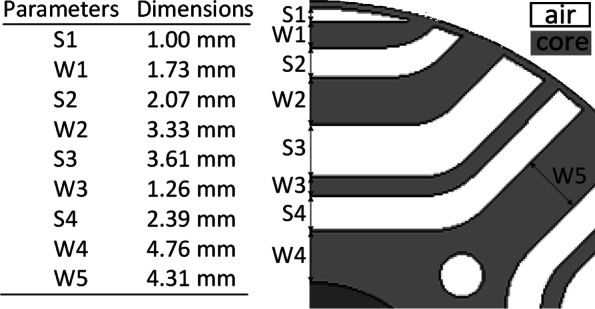
Optimized rotor parameters and quarter view of rotor.

Fig. 4 presents the optimized rotor parameters along with a quarter view of the rotor. The table lists the dimensions of various segments, identified as S1 through S4 and W1 through W5. These parameters are critical in defining the rotor geometry and ensuring optimal performance. The accompanying diagram visually represents these parameters on a quarter section of the rotor. The distinctions between air and core regions are clearly marked, showing the specific locations of each parameter. The precise dimensions listed contribute to the overall efficiency and performance of the rotor, indicating a meticulous optimization process. The optimization resulted in a motor torque of 1.76 Nm and a torque ripple of 11.713% at full load. The optimal rotor geometry dimensions are detailed in Fig. 4, with an inner radius of 10 mm and an outer radius of 29.5 mm. During optimization, the dimensions were constrained to a maximum of 5 mm and a minimum of 0.05 mm, while all other rotor dimensions were kept constant.

**Figure 5:**
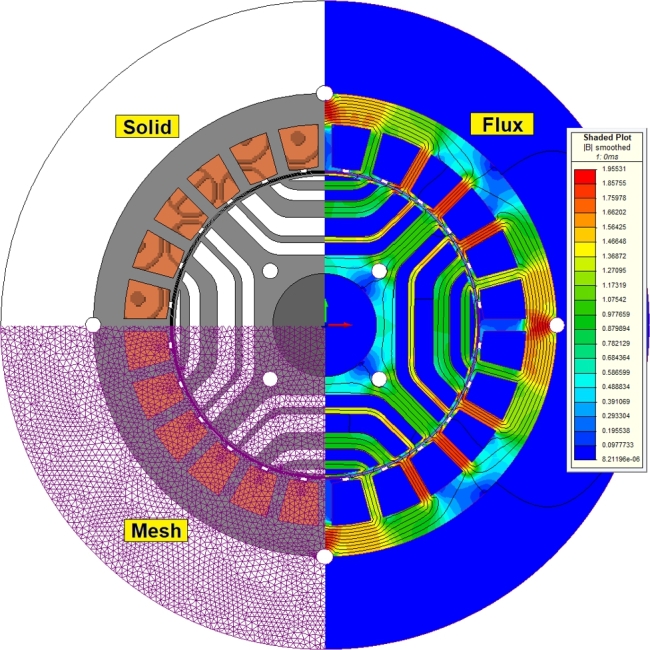
Solid, mesh and flux lines views of optimized motor.

**Figure 6:**
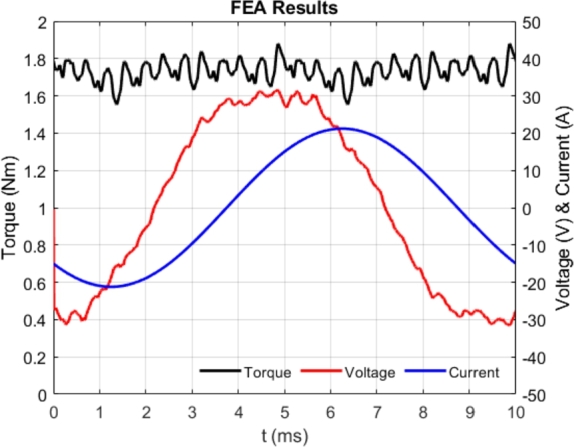
Results of FEA for torque, voltage, current waveforms.

The optimized motor structure is displayed in Fig. 5, showcasing solid, mesh, and flux line views. As depicted, the flux lines are appropriately distributed and oriented within the rotor's flux paths. The torque generated by the optimized motor, analyzed using MAGNET-based FEA, is illustrated in Fig. 6. The motor was operated for two mechanical cycles over 10 ms, with the electrical waveforms shown as one cycle in the figure. The torque fluctuated between 1.6 Nm and 1.8 Nm. The motor windings were powered by a current source, as indicated in the figure. The voltage on the A-phase winding exhibited low harmonic content, as shown in Fig. 6. The phase voltage and phase current had an angle of approximately 55 electrical degrees. This suggests that SynRMs generally have a lower power factor compared to other AC motors.

Table 2 presents the lumped parameters of the designed motor, which include key electrical characteristics essential for its performance analysis. The Back-EMF (Electromotive Force) is 0 V indicating that no induced voltage is generated when the motor is rotating. The torque constant (Kt), derived from the back EMF constant (Ke), is 0 N⋅m/A, reflecting the same operational state. The q-axis inductance (Lq) and d-axis inductance (Ld) are 1.0925 mH and 3.7731 mH, respectively. These values are crucial for understanding the motor's reactance in different axes, impacting its dynamic response. The ratio of Ld to Lq is 3.4534, showing the relationship between the aligned and unaligned inductances. The minimum per phase self-inductance (Lmin) and maximum per phase self-inductance (Lmax) are 0.9452 mH and 2.6810 mH, respectively, which are significant for analyzing the variation in inductance due to the rotor position. Lastly, the stator phase resistance (Rs) is 0.1321 Ω, a vital parameter affecting the power losses and efficiency of the motor. Together, these parameters provide a comprehensive overview of the designed motor's electrical properties, serving as a foundation for further analysis and optimization.

**Table 2:** Lumped parameters of designed motor.

Parameters	Values
*Back-EMF (peak line-line)*	0 V
*Kt (derived from Ke)*	0 N⋅m/A
*Lq (q-axis inductance)*	1.0925 mH
*Ld (d-axis inductance)*	3.7731 mH
*Ld/Lq (aligned/unaligned)*	3.4534
*Lmin (minimum per phase self-inductance)*	0.9452 mH
*Lmax (maximum per phase self-inductance)*	2.6810 mH
*Rs (stator phase resistance)*	0.1321 Ω

**Figure 7:**
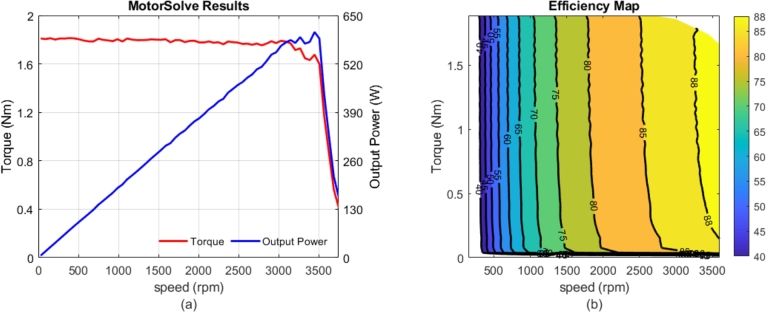
a) Curves of torque and output power, b) Efficiency map.

The optimized motor was analyzed by using MotorSolve program, as shown in Fig. 7. Fig. 7(a) presents the curves for the optimized motor's output power and torque. As indicated, the motor was capable of producing approximately 1.8 Nm of torque from zero speed to its nominal speed and delivered the desired output power of around 0.55 kW at rated speed. Additionally, the efficiency map of the motor is displayed in Fig. 7(b). It shows that the motor achieves over 80% efficiency at speeds above 2000 rpm and approximately 87% efficiency at its nominal operating condition.

## 5 Analysis of proposed system with solar PV panels

**Figure 8:**
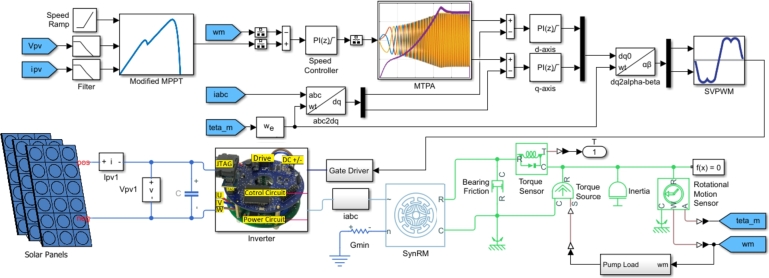
Proposed system with solar PV panels.

The proposed system was analyzed by connecting it to solar PV panels. The simulation of the developed system is illustrated in Fig. 8. MATLAB/SimScape blocks were used to achieve nearly realistic results in the simulation environment. A modified MPPT algorithm, as detailed in [14], was employed to maximize the power extracted from the panels. The modified P&O MPPT algorithm for speed control is given in Algorithm-1. The provided algorithm outlines a decision-making process based on changes in voltage and power over time, commonly used in MPPT for optimizing power output. It begins by taking in several inputs: the current and previous values of voltage (V(t), V(t−Δt)), current (I(t), I(t−Δt)), and power (P(t), P(t−Δt)). It then calculates the changes in voltage (Δ*V*) and power (Δ*P*) over the time interval Δ*t*. The algorithm first checks if the change in power (Δ*P*) is zero. If it is, the process terminates and returns, implying no adjustment is needed. If Δ*P* is positive, the algorithm assesses the change in voltage (Δ*V*). If Δ*V* is positive, it decreases the reference value $n_{\text{ref}}$; otherwise, it increases $n_{\text{ref}}$. Conversely, if Δ*P* is negative, the same conditions apply: it decreases $n_{\text{ref}}$ if Δ*V* is positive, and increases $n_{\text{ref}}$ if Δ*V* is negative. This logic ensures that the system continually adjusts to maintain optimal performance based on the observed changes in power and voltage. The process concludes by returning the final state.

**Algorithm 1:**
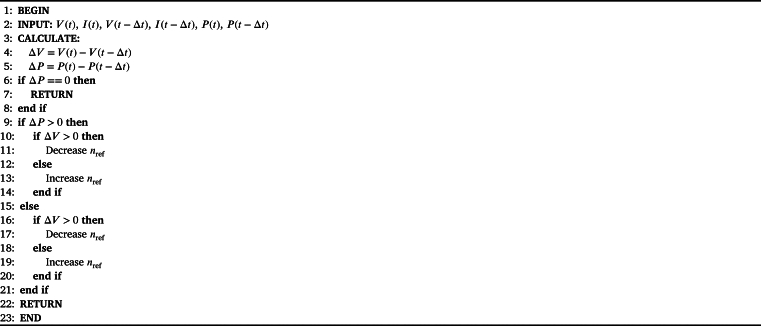
Modified P&O MPPT algorithm for speed control.

The MPPT algorithm requires the DC-link voltage, obtained from the terminals of the solar PV panels. Additionally, a ramp reference was used to initiate the motor. The MPPT block determines the motor reference speed to obtain maximum power. The MTPA algorithm controls the *dq*-axis voltages to generate the PWM signals, creating a closed loop for controlling the SynRM directly connected to the solar PV panels. The SynRM drives a pump load, as shown in Fig. 8. The pump load was simulated using Eq. (9). $\omega_{motor}$ indicates the actual speed of the motor during the operation, while $\omega_{rated}$, determined as 3000 rpm, denotes the motor's synchronous speed.

(9) $$T_{\begin{array}{c} pump\\ load \end{array}}={\left(\frac{\omega_{motor}}{\omega_{rated}}\right)}^{2}T_{rated}$$

**Figure 9:**
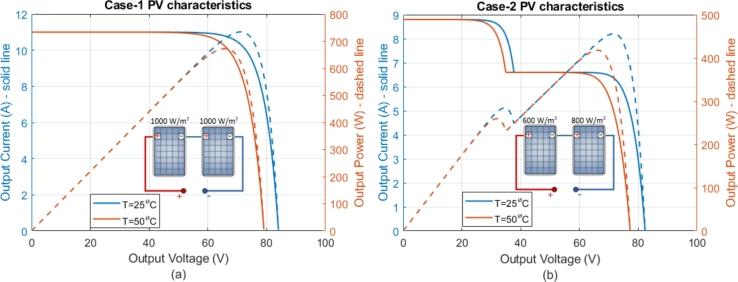
Solar PV characteristics; a) PV1=1000 W/m^2^ + PV2=1000 W/m^2^, b) PV1=600 W/m^2^ + PV2=800 W/m^2^.

**Figure 10:**
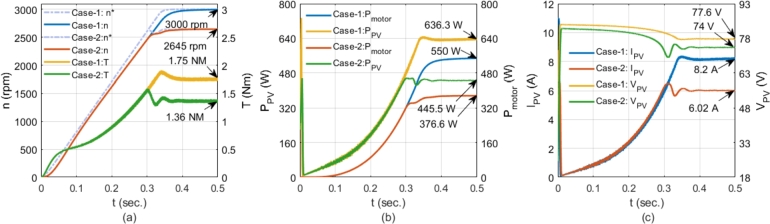
Analysis for Case-1 and Case-2 conditions; a) Motor speed and motor torque, b) Power of motor and PV panels, c) PV Current and PV voltage.

Two solar PV panels were used to test the system's performance under different conditions. Each panel, consisting of 60 cells, can generate 375 W with an efficiency of 20.7% (solar PV panel model: LG NeON® 2). The panels were connected in series, with each panel having an open-circuit voltage of 42 V and a short-circuit current of 11 A at standard test conditions (STC). Two scenarios were created: full irradiation and partial shading. In Case-1, the irradiation for both panels was set to 1000 $\text{W}/{\text{m}}^{2}$, as shown in Fig. 9a. In Case-2, partial shading was simulated by adjusting the first panel to 600 $\text{W}/{\text{m}}^{2}$ and the second to 800 $\text{W}/{\text{m}}^{2}$, as depicted in Fig. 9b. In Case-1, the power provided by the panels exceeded the motor's power requirements. In Case-2, the power generated was less than the motor's output power. The motor was operated under the conditions for both cases. The performance results of the proposed system are shown in Fig. 10. For Case-1, where the motor operated at rated values, the conditions are colored blue and yellow in Fig. 10. In Case-2, where the motor operated below rated values, the conditions are colored red and green. In Case-1, the power generated from the solar PV panels was higher than the motor's power requirement, allowing the motor to run at 3000 rpm with 1.75 Nm of torque, as shown in Fig. 10a. In Case-2, where power was lower than the motor's rated power, the motor's speed and torque were reduced to 2645 rpm and 1.36 Nm, respectively, due to MPPT control, as shown in Fig. 10a. The output power of the solar PV panels and the motor is presented in Fig. 10b for both cases. The system efficiencies were 86.4% for Case-1 and 84.53% for Case-2, with inverter losses neglected in these results. Fig. 10c shows the currents and voltages of the solar PV panels for both cases. The effect of MPPT can be observed within 0.3 seconds, during which the currents were fixed to maximize power. The simulation results demonstrate that the proposed system is suitable for direct connection to solar PV panels without additional devices and can operate with two solar PV panels connected in series.

## 6 Prototyping of motor and integrated motor drive

**Figure 11:**
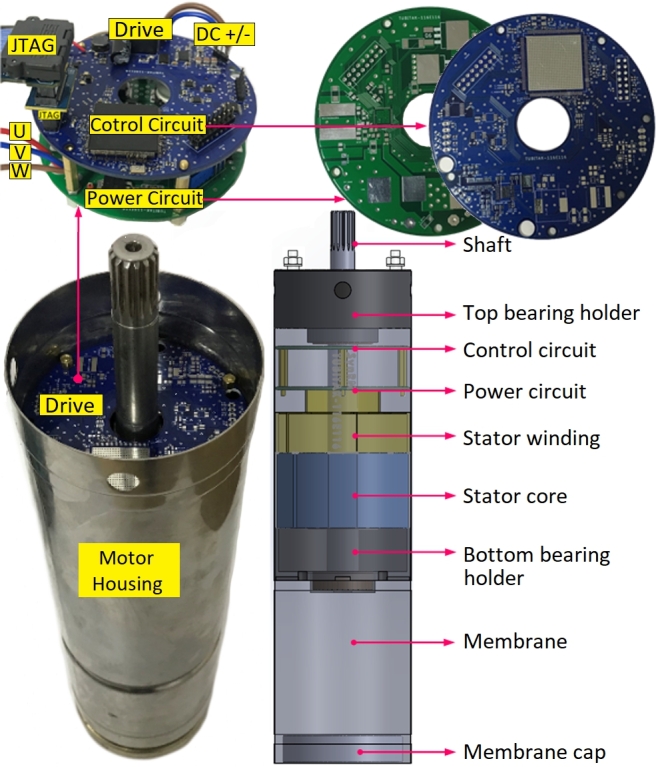
Prototype of motor and integrated drive.

The proposed system was designed for solar water pumping applications, using a commercial 4-inch submersible pump motor case as the housing for the prototype motor. Initially, the stator and rotor cores were produced using a laser cutting machine. Four stud bolts were employed to assemble the rotor laminations, while the stator laminations were assembled using a cold welding process. This approach created a rigid structure for both the stator and rotor stacks. In mass production, however, this procedure could be replaced with a locking lamination die for efficiency. Subsequently, the stator, rotor, and shaft were assembled into the 4-inch submersible pump motor housing, as shown in Fig. 11. Additionally, a motor drive circuit was designed for this motor, which consists of two distinct PCBs: the power circuit and the control circuit, as illustrated in Fig. 11. The power circuit includes an inverter structure, utilizing IRFS4115PBF MOSFETs and a DRV8353RH gate drive IC. The control circuit features a 32-bit floating-point MCU, the TMS320F28379D. This MCU has dual CPU cores: one dedicated to executing the motor control algorithm and the other to the sensorless algorithm. This separation allows for a low sample time and efficient motor control.

**Figure 12:**
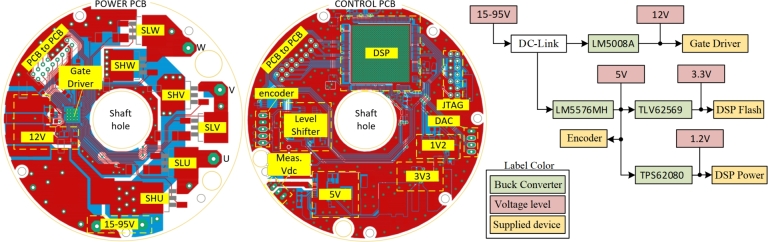
Designed power and control PCBs.

A detailed view of the designed PCBs is shown in Fig. 12. The MOSFET switches are positioned on the right-hand side of the power PCB, with *SLx* and *SHx* denoting the lower and higher sides associated with the inverter's phase arm, respectively. The three-phase motor connections are labeled *U*, *V*, and *W*. On the left-hand side of the board, the gate driver IC and a 12 V buck-converter are located. The gate driver IC includes an integrated buck-converter IC, which requires only passive components for operation. The control PCB is designed separately from the power circuit to minimize susceptibility to electromagnetic interference (EMI) and is connected to the power circuit via *PCB-to-PCB* header pins, as illustrated in Fig. 12. The magnetic field in the upper and lower regions of the stator is minimal. Additionally, the motor case is made of stainless steel, which reduces magnetic interference reaching the PCB and significantly mitigates EMI issues. The proposed motor benefits from superior heat transfer compared to traditional surface-mounted motors due to its submersible design, which operates in cooler underwater environments. Moreover, SynRM motors experience less heating since they lack rotor copper losses. These motors are also referred to as oil-cooled submersible pump motors because oil within the motor case aids in cooling. This design ensures effective heat dissipation for both the motor and the motor drive circuit. The control PCB features a DSP, a JTAG connection, and three different voltage-level buck converters. Due to the DC-link voltage range of 15 V to 95 V, standard voltage regulators are inadequate. The system requires 1.2 V for the DSP Vdd power, 3.3 V for the DSP flash memory, 5 V for the encoder and other ICs, and 12 V for the gate drive IC. Consequently, four buck converters are used to obtain the necessary voltage levels from the DC-link voltage. The block diagram of the voltage levels and device names is shown in Fig. 12. The buck converters use commercial ICs, including the LM5008A, LM5576MH, TLV62569, and TSP62080.

## 7 Experimental study

**Figure 13:**
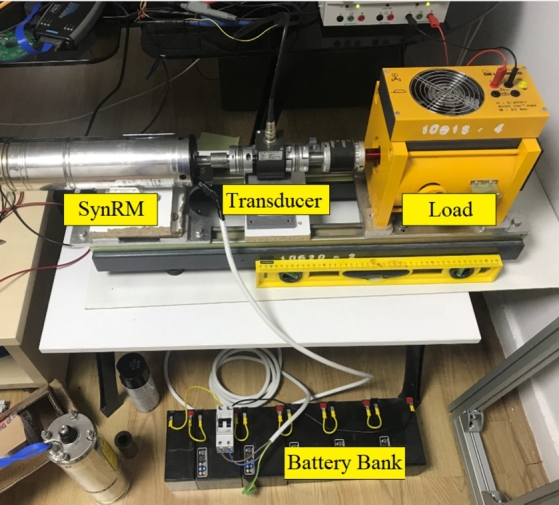
Experimental setup.

**Figure 14:**
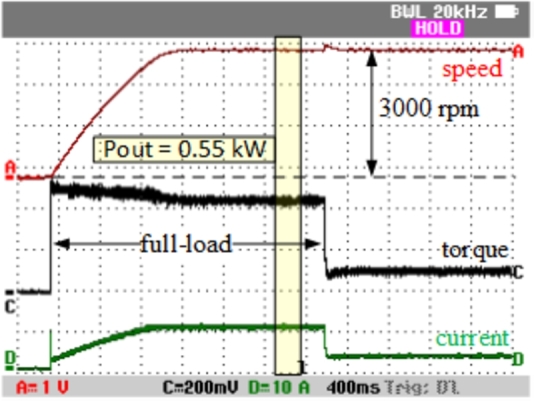
Experimental results for nominal operation.

The experimental setup is shown in Fig. 13. The motor, labeled as SynRM in the figure, was tested using a magnetic powder brake as the load. Both the submersible pump motor and the motor drive were evaluated under full load conditions, as depicted in Fig. 13. Due to the laboratory power source's inability to provide the necessary power for nominal operation, a 6-battery stack was used to power the system. The motor was tested at a full load value of 1.75 Nm and operated at the nominal speed of 3000 rpm, as illustrated in Fig. 14. In Fig. 14, Channel-A displays the motor speed, Channel-C shows the motor torque, and Channel-D represents the DC-link current. During operation at 3000 rpm with a 1.75 Nm load, the system drew approximately 11 A of current from the DC-link. When the load was removed, the current decreased to approximately 0.35 Nm, which corresponds to the idle load of the magnetic brake.

The motor's acceleration and load transition response are quite satisfactory for pump applications. Currently, there are no commercially available submersible pump motors of the SynRM type, so a direct comparison with existing SynRM models is not possible. A comparison of the prototype motor with commercial submersible pump motors is provided in Table 3. Induction motors (IMs) and brushless DC (BLDC) motors are commonly used in solar-powered water pumping systems. IMs typically require boost converters due to their inability to operate at low voltage levels. The prototype motor outperforms IMs in several rated parameters. Although it slightly lags behind BLDC motors, this is outweighed by its superior price-performance ratio. Additionally, the SynRM's rotor structure offers greater durability compared to other motor types.

**Table 3:** Comparison of prototype motor with commercial submersible pump motors.

Rated	Prototype SynRM	Induction Motor	BLDC
*Power (W)*	550	550	700
*Speed (rpm)*	3000	2835	3300
*Voltage (V)*	48	380	60
*Current (A)*	15	2	13
*Power Factor*	0.51	0.63	-
*Efficiency (%)*	86.48	66.32	90
*Cost*	low	medium	high
*Durability*	high	medium	low

Experimental and simulation studies can generally be evaluated based on measurement accuracy. Since control is performed by measuring the stator currents of the motor, it is crucial to measure the current signals accurately. Both sensor sensitivity and calibration are important during current measurement. In this study, the ACS758LCB-050B current sensor is used. This sensor is capable of bidirectional measurement with a sensitivity of 40 mV/A. The DSP used can measure analog signals with 12-bit conversion in as little as 280 ns. Due to these values, no issues were observed. However, it is impossible to produce the optimized geometry of the motor design flawlessly. Therefore, an efficiency difference of approximately 0.5% to 1.5% was measured between the FEA-analyzed motor results and the actual motor. This difference can be considered within acceptable limits. The reasons for this difference can be attributed to the material properties of the laminated sheet, manufacturing precision, and measurement errors. Additionally, the viscosity of the cooling oil inside the motor, which can partially affect friction losses, should be considered. When the proposed system operates with a real PV panel, the PV panel may not have the exact same characteristics as the PV panel in the simulation environment. This discrepancy should be taken into account.

## 8 Conclusion

Electric motors, particularly pump motors, account for a significant portion of electrical energy consumption in industrial applications. Enhancing these motors can substantially impact overall energy use. This study presents a designed and optimized SynRM pump motor and motor drive system tailored for small-scale irrigation systems, aiming to maximize solar energy utilization. The SynRM was selected for its high efficiency, durability, and cost-effectiveness. The motor was engineered to minimize torque ripple and maximize torque output, specifically for low voltage levels typical of solar PV panels, eliminating the need for a boost converter. With a power output of 0.55 kW and an efficiency of 86.5%, the motor meets the ultra-premium efficiency standards set by IEC TS 60034-30-2:2016, making it highly suitable for small-scale solar pumping systems.

The motor, designed as a 4-inch submersible pump motor, features a specially engineered drive system that fits within the motor housing. This integrated motor drive system includes separate power and control circuits and can be directly powered by PV panels, avoiding the need for additional devices. This results in a more compact structure compared to traditional systems. This study highlights the feasibility of achieving a high-efficiency, low-voltage motor with a compact integrated drive system that can be directly powered by solar panels, providing a significant alternative to existing solutions.

As a future work, it might be possible to achieve higher power density by incorporating non-rare magnets into the rotor as an alternative to the motor presented here. This would allow for a higher motor power within the same dimensions. In this study, the proposed motor driver is designed to be placed on the drive shaft side of the motor, requiring the PCB board to be drilled in the center to accommodate the shaft diameter, which introduces certain limitations. In future studies, a special area could be created between the lower bearing seat and the pressure membrane of the submersible pump motor to place the driver. Additionally, it is possible to work on control algorithms focused on minimizing torque ripple to reduce motor torque oscillations.

## CRediT authorship contribution statement

**Gullu Boztas:** Writing – original draft, Software, Methodology, Investigation. **Omur Aydogmus:** Writing – original draft, Validation, Formal analysis, Conceptualization. **Musa Yilmaz:** Writing – review & editing, Validation, Supervision.

## Declaration of Competing Interest

The authors declare that they have no known competing financial interests or personal relationships that could have appeared to influence the work reported in this paper.
